# Evaluation of Fossil Amber Birefringence and Inclusions Using Terahertz Time-Domain Spectroscopy

**DOI:** 10.3390/polym14245506

**Published:** 2022-12-15

**Authors:** Alexander T. Clark, Sophia D’Anna, Jessy Nemati, Phillip Barden, Ian Gatley, John Federici

**Affiliations:** 1 Department of Physics, New Jersey Institute of Technology, Newark, NJ 07102, USA; 2 Federated Department of Biological Sciences, New Jersey Institute of Technology, Newark, NJ 07102, USA; 3 Division of Invertebrate Zoology, American Museum of Natural History, New York, NY 10024, USA

**Keywords:** terahertz, stress, birefringence, amber, photoelasticity, nondestructive evaluation

## Abstract

Using a cross-polarization transmission geometry, stress maps for the normalized birefringence and intrinsic stress direction of polymeric materials may be obtained using terahertz nondestructive evaluation. The analysis method utilizes a deconvolution method to determine the arrival times and amplitude of the cross-polarized terahertz pulses through a birefringent material. Using amber (a naturally occurring polymer) as a material of interest, stress maps show that inclusion-free Lebanese amber samples behave as classic uniaxial birefringent (photoelastic) materials whose principal stress directions, as inferred in the terahertz spectral range, agree well with visible photoelasticity measurements. Since amber samples, depending upon their source, may be either transparent or opaque to visible light, comparing birefringence measurements in the visible and terahertz spectral ranges cross-validates the stress measurements, thereby establishing a strong and unique stress analysis methodology for visibly opaque samples. While the material of interest for this paper is amber, the method is generally applicable for any terahertz-transparent polymer. The cross-polarization experimental configuration enables stress levels within the amber matrix to be visualized while also outlining highly localized regions of stress surrounding inclusions. Birefringence stress maps clearly show localized increases in stress magnitude and directional changes surrounding inclusions.

## 1. Introduction

Over the past twenty years, terahertz (THz) imaging and spectroscopy has been applied to the nondestructive evaluation (NDE) of materials [1]. Terahertz wavelengths (3 mm to 100 μm) are in the electromagnetic spectral band between microwave and infrared light. Recent NDE applications include composite structures [2], additive manufacturing [3], the agricultural-food industry [4,5,6], pharmaceutical products [7], and moisture monitoring [8]. Terahertz radiation may be used for the nondestructive imaging of materials such as plastics, ceramics, and other dielectrics since these materials tend to exhibit relatively small absorbance in the terahertz range, enabling the imaging of the internal structure as well as defects/ inclusions. Terahertz polarimetry and birefringence instrumentation based on polarization-sensitive photoconductive detectors and electro-optic detection of the polarization state have been developed [9,10,11,12,13]. Recent examples of terahertz birefringence measurements for the nondestructive evaluation of polymers include polymer liquid crystals [14], injection-molded thermoplastics and welds [15,16] as well as mechanical stress in stretched poly(lactic acid) (PLA) films [17]. This paper emphasizes terahertz nondestructive measurement methodologies to determine the intrinsic stress birefringence of polymer materials and the imaging of inclusions and focuses on the detection of inclusions in naturally occurring amber. Since amber samples, depending upon their source, may be either transparent or opaque to visible light, comparing birefringence measurements in the visible and terahertz spectral ranges cross-validates the stress measurements, thereby establishing a strong and unique stress analysis methodology for visibly opaque samples. While the material of interest for this paper is amber, the method is generally applicable for polymers and any terahertz-transparent material.

Amber derives from fossilized plant resin and uniquely preserves aspects of ancient ecosystems. Plants exude resins in response to damage. These resins, when subjected to heat and pressure over extended periods of time, undergo polymerization that leads to fossilization [18]. Biotic or abiotic material that comes into contact with fresh resin may ultimately become entombed within amber; these entombed elements are termed inclusions. Organic inclusions can range from vertebrate remains to microscopic elements including single-celled protists [19,20], while notable inorganic inclusions span putatively ancient gas and liquid [21,22]. There are many dozens of amber deposits worldwide, the oldest of which are ~320 MegaAnnum (Ma) in age [23]; however, the oldest known inclusions date to the Triassic ~230 Ma [24]. The majority of known amber samples are dated to the last 100 million years, particularly in the Cenozoic 65 Ma to the present day [25]. 

Amber-based research focuses primarily on the description and characterization of organic inclusions. Amber is frequently optically transparent, and as such light microscopy is most often employed in sample analysis. However, other bands of electromagnetic radiation are used to resolve inclusions and determine the provenance and material properties of amber itself. Techniques outside of the visible light spectrum include confocal laser scanning microscopy (CLSM) [26], infrared (IR) and Fourier transform infrared (FTIR) spectroscopy [27], nuclear magnetic resonance (NMR) [28], X-ray based CT-scanning [29], and, recently, terahertz spectroscopy (THz) [30,31]. 

While light microscopy, X-ray imaging, Raman spectroscopy, and infrared spectroscopy [32] remain the most common methods in amber research, the terahertz spectral range has recently been adapted to spectroscopically characterize amber, detecting the presence of inclusions and differentiating between real and fake amber [31]. In our prior work, amber samples representing a variety of ages, botanical sources, and geographic locations were characterized using terahertz spectroscopy. Time of flight terahertz measurements through amber demonstrate that for samples without inclusions from the same deposit, the measured refractive indices of transparent samples are near equal. There is no obvious trend that relates the sample age or botanical source (at a higher taxonomic level) to the real refractive index across measured samples. The introduction of inclusions into the amber (including turbidity or minerals mixed in with the amber) generally will cause the refractive index to increase relative to ‘clear’ amber samples. A comparison of the measured permittivity, real refractive index, and attenuation of counterfeit amber shows that terahertz spectroscopy can easily distinguish between a fraudulent specimen derived from at least some synthetic resins and amber. 

Here, the application and utility of terahertz birefringence in the characterization of amber material properties and the detection of inclusions in amber is investigated. Because fossil specimens, particularly those relating to the description of new species (holotypes), are irreplicable and frequently trimmed to better visualize and conserve specimens [33], the mapping of the intrinsic strain within amber samples via terahertz birefringence may serve to identify specimen locations within the amber and aid in sample trimming without destroying potentially valuable specimens. Moreover, the potential of terahertz birefringence imaging in resolving inclusions within amber is explored through terahertz-computed tomography (THz-CT). Such a technique would provide an additional dimension in three-dimensional imaging to compliment current X-ray-based approaches. Fossil samples ranging from ~16 to 119 Ma from the Dominican Republic, Mexico, and Lebanon are assessed. The presence of insect inclusions is imaged using the birefringence of amber resulting from internal stress as well as THz-CT.

## 2. Materials and Methods

### 2.1. Measurement of Internal Stress/Birefringence of Amber

In a homogeneous material, the speed at which an electromagnetic wave propagates through the material (as measured by the material’s refractive index) is independent of the polarization direction of the wave’s electric field direction. However, it is well known that when mechanical stress is present (either intrinsic or applied stress), the refractive index of the material depends on the polarization of the wave relative to the stress direction present within creating what is known as a photoelastic material [34]. In its simplest form, the photoelasticity is manifested by a double refracting or birefringent material: electromagnetic waves polarized parallel to the stress direction experience one refractive index, while waves polarized perpendicular to the stress direction experience a different refractive index. Stress direction is represented as a line or plane, parallel with the fast axis, in which stress is present within the material in relation to a laboratory axis.

Since amber precursors are exuded from plants, there is a flow associated with their creation. This flow, as the materials polymerize over time, will create a birefringence within the resulting amber due to varying levels of stress within, creating a photoelastic material. Birefringence induces a relative phase difference between waves polarized parallel to and perpendicular to the stress direction. In this paper, the measurement of the intrinsic birefringence and stress of amber samples is emphasized. This measurable birefringence can aid in both the understanding of the origins of the amber samples as well as in the detection of inclusions since intrinsic stress tends to concentrate at the inclusions, both in the visual and terahertz spectral ranges. Measuring the birefringence can also allow for the determination of the direction of the stress within the amber, which can further be used to obtain insight into the flow and origin of the amber measured. Prior measurements on birefringence from amber inclusions show that birefringence in polarized visible light is a property of certain organic inclusions. Setae, which are found in arthropods and are analogous to hairs found in vertebrates, exhibit birefringence. Because setal composition is used in the identification of certain groups of organisms, measurable birefringence has been used to identify amber inclusions, for example in oribatid mites [35].

The primary aim of this study was to describe the birefringent properties of amber with and without inclusions. The secondary aim of this study was to use an index-matching liquid to remove the refractive effects of the air–amber boundary. A major complication that terahertz imaging faces is refraction at the boundaries of materials due to differences in refractive index. This refraction can radically change the direction of wave propagation, thereby violating the parallel ray projection approximation which is typically assumed in tomographic image reconstruction [36]. Index-matching materials can be used to reduce this refraction, allowing for more precise and accurate images. Index-matching materials are usually liquids or gels that have a similar real refractive index to the object of interest. When submerged, the index-matching material allows light to pass through from the index-matching material into the sample with minimal reflection or refraction. This technique has been applied previously to THz imaging using liquid paraffin and benzocyclobutene (BCB) in an attempt to create a custom-characterized index-matching fluid for 2D and 3D imaging [37,38,39]. Applying this methodology to amber samples allows for faster and more accurate imaging of the specimen while also increasing the accuracy and decreasing the time needed for inclusion detection.

### 2.2. Amber Samples

Four amber samples were used in this study. The first sample (AMNH-Bal_Mex1_B) was from Chiapas, Mexico and was deposited during the Miocene 16 million years ago (Ma). Sample AMNH-Bal_Mex1_B had been trimmed and polished so that it had two opposing and approximately parallel faces. The second sample (AMNH-Bal_Leb1_C) was from Bcharre, Lebanon, was deposited during the Early Cretaceous 125–129 Ma and was used for visual and THz birefringence imaging. The third amber sample (BALDR_0076) was from the Dominican Republic, was also dated to the Miocene 16 Ma, and was used for THz-CT both for its distinct shape and present inclusions in the form of two termite workers from the species Mastotermes electrodominicus. The fourth sample (BALDR_0239) was also from the Dominican Republic and was chosen for polarization imaging analysis due to the large number of bubbles which are trapped in the amber. The amber samples were typically mounted to various standard optical posts using dental wax.

### 2.3. Terahertz Instrumentation

The samples were imaged and spectroscopically characterized using a terahertz time-domain spectroscopy system. In this methodology, short time-duration (several picoseconds) electromagnetic pulses are generated and detected using optoelectronic techniques [40,41]. Using a T-Ray 5000 terahertz time-domain system (Luna TeraMetrix, Ann Arbor, MI, USA), terahertz time-domain waveforms were acquired in a transmission mode through the sample for a 160 ps time window. The terahertz time-domain waveforms were analyzed as described below to extract the stress angle and relative magnitude of the stress-induced birefringence. Images were acquired on a pixel-by-pixel basis by scanning the sample through the beam path. 

**Figure 1:**
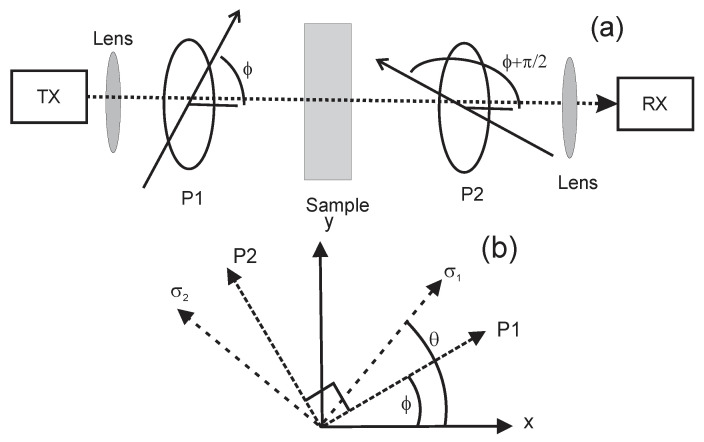
Experimental configuration for measurement of birefringence in a transmission mode. (**a**) relative positions of the transmitter/polarizer P1 module, birefringent sample, and receiver/polarizer P2 module. (**b**) Orientation of transmission axes of polarizers P1 and P2 relative to the laboratory x and y axes. The terahertz pulse is propagating into the page. The fast $\sigma_{1}$ and slow $\sigma_{2}$ axes of the birefringent material are assumed to be orthogonal.

An illustration of the experimental setup is shown in Figure 1. The terahertz transmitter and receiver units for the T-Ray 5000 system were linearly polarized. Terahertz radiation was focused onto the sample using 3-inch focal length lenses. The polarizer P1 was attached to the terahertz transmitter so that the polarization transmission axes for both the transmitter and polarizer are the same. The receiver module and polarizer P2 were also aligned with the same transmission axes and rotated as a unit. Figure 1b illustrates the orientation of the transmission axes of polarizers P1 and P2 relative to the laboratory x and y axes as well as the orthogonal fast $\sigma_{1}$ and slow $\sigma_{2}$ axes of the birefringent material. For reference, the terahertz pulse was propagating into the page. The birefringence analysis method was based on a simple crossed polarizer configuration, as introduced by Kang et al. [42]. Rather than analyzing the polarization properties of the transmitted terahertz waves using a Jones matrix approach, here, an alternative method was used which is more amenable to time-domain analysis. Mathematical details may be found in Appendix A.

## 3. Results

For simplicity, it was assumed that the sample can be treated as a uniaxial birefringent material in which the principal stress axes are perpendicular to the propagation direction of the terahertz waves. Clearly, this assumption may not always be true for amber samples considering the potential for variable resin flows and/or stresses which may be present during polymerization. However, as will be shown for the Lebanese amber samples, the observed birefringence behavior was consistent with the simplifying assumption of a uniaxial birefringent material. 

The electric field after transmission through polarizer P1 in Figure 1 can be expressed as

(1) $$E_{TX}=E_{inc}\left(t\right)\left(\hat{x}\cos\phi +\hat{y}\sin\phi \right)$$

where ϕ is the angle between the horizontal x-axis and the transmission axis of polarizer P1. As detailed in Appendix A, the stress direction may be determined by

(2) $$\frac{E_{o}}{E_{45}}=\frac{2\sin\theta \cos\theta }{\sin^{2}\theta -\cos^{2}\theta }=-\tan2\theta$$

The amplitudes $E_{0}$ and $E_{45}$ denote the amplitudes of the measured time-domain waveform for the polarizer P1 making angles of ϕ = 0° and ϕ = 45° with respect to the x-axis (polarizer P2 is orthogonal to P1 for both values of ϕ). The magnitude of the birefringence $\Delta n=n_{\sigma_{2}}-n_{\sigma_{1}}$ may be expressed as

(3) $$\frac{\Delta n}{n_{average}-1}=2\frac{\Delta t_{o}}{\left(\Delta t_{\sigma_{1}}+\Delta t_{\sigma_{2}}\right)/2}\sqrt{{\left(\frac{E_{o}}{\tau_{as}\tau_{sa}E_{diff}^{}}\right)}^{2}+{\left(\frac{E_{45}}{\tau_{as}\tau_{sa}E_{diff}^{}}\right)}^{2}}$$

with

(4) $$\tau_{as}\tau_{sa}E_{diff}\left(t\right)=\tau_{as}\tau_{sa}\left(E_{inc}\left(t+\left(\Delta t_{\sigma_{1}}+\Delta t_{\sigma_{2}}\right)/2\right)-E_{inc}\left(t+\left(\Delta t_{\sigma_{1}}+\Delta t_{\sigma_{2}}\right)/2+\Delta t_{o}\right)\right)$$

The propagation times for terahertz pulses traveling along the two principal stress axes in the material are given by $\Delta t_{\sigma_{1}}=n_{\sigma_{1}}L/c_{o}$ and $\Delta t_{\sigma_{2}}=n_{\sigma_{2}}L/c_{o}$. The refractive indices along the two principal axes are denoted by $n_{\sigma_{1}}$ and $n_{\sigma_{2}}$. In the above equations, *L* is the sample thickness, $c_{o}$ is the speed of light in a vacuum, and $\Delta t_{o}$ is the time step in the digitized time-domain waveform. The product $\tau_{as}\tau_{sa}$ accounts for any losses in transmission due to reflection from the air–sample and sample–air interfaces. The differential electric field pulse $E_{diff}$ is generated mathematically by subtracting the incident electric field waveform shifted in time by $\Delta t_{o}$ from the incident waveform. For simplicity, a value of $\Delta t_{o}$ = 0.1 ps, which is the time interval in the terahertz waveforms digitized by the T-Ray 5000 system, was used.

### 3.1. Experimental Determination of Pulse Amplitudes and Time Shifts

The extraction of the stress direction from Equation (2) and the relative birefringence from Equation (3) requires the measurement of four terahertz pulse waveforms. The time-domain waveforms through the sample with polarizers P1 and P2 oriented orthogonally, as illustrated in Figure 1 with ϕ = 0° and ϕ = 45°, correspond to $E_{o}$ and $E_{45}$. The measured waveform through the sample with P1 and P2 oriented parallel to each other corresponds to $\tau_{as}\tau_{sa}E_{inc}\left(t+\left(\Delta t_{\sigma_{1}}+\Delta t_{\sigma_{2}}\right)/2\right)$. The last required waveform is the one with the sample removed, which measures $E_{inc}\left(t\right)$. The waveform $E_{diff}^{}$ is calculated by shifting the $E_{inc}\left(t\right)$ waveform by one time slot (corresponding to $\Delta t_{o}$ = 0.1 ps for the Terametrix T-Ray 5000 system) and subtracting the shifted waveform from the unshifted waveform. 

The amplitude and arrival time for each of the four waveforms was mathematically determined by a deconvolution process using the T-Ray 5000 software. The deconvolution was performed in the frequency domain using three filters. The first was a Gaussian filter in the time-domain with a 0.8 ps bandwidth. The second was a Bessel bandpass filter from 0.2–0.8 THz. The last filter was a Wiener filter. The arrival time $\left(\Delta t_{\sigma_{2}}+\Delta t_{\sigma_{1}}\right)/2$ was calculated by deconvolving the waveform $\tau_{as}\tau_{sa}E_{inc}\left(t+\left(\Delta t_{\sigma_{1}}+\Delta t_{\sigma_{2}}\right)/2\right)$ with $E_{inc}\left(t\right)$ as the reference. The amplitudes of the $E_{o}\left(t\right)$ and $E_{45}\left(t\right)$ waveforms were calculated by deconvolving the respective waveforms with $E_{diff}^{}$.

**Figure 2:**
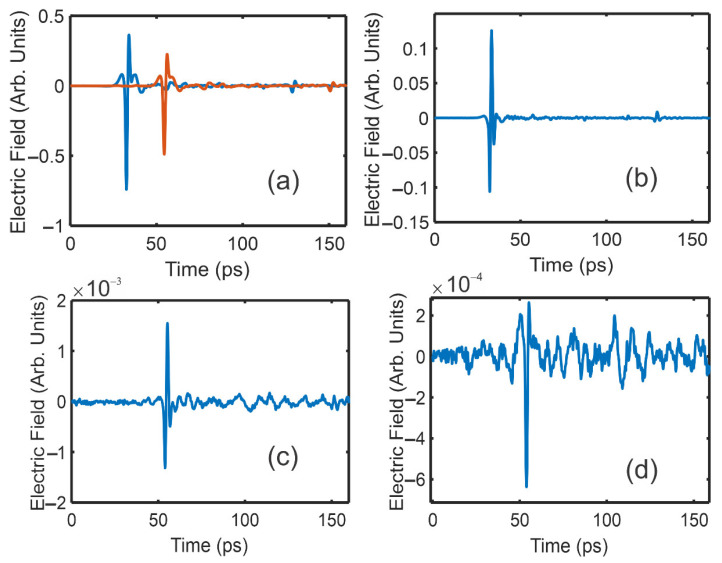
Measured terahertz waveforms for (**a**) $E_{inc}\left(t\right)$ (blue curve) and $\tau_{as}\tau_{sa}E_{inc}\left(t\right)$ (red curve). The time delay between the pulses is a measurement of the optical path length through the sample. (**b**) $E_{diff}^{}\left(t\right)$ waveform (**c**) $E_{o}\left(t\right)$, and (**d**) $E_{45}\left(t\right)$.

**Figure 3:**
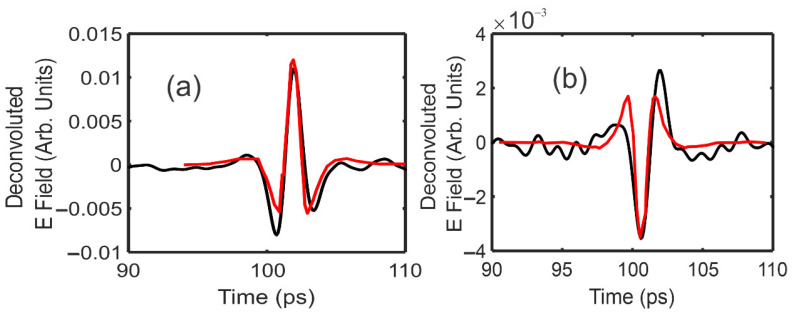
Deconvoluted terahertz waveforms (black) and best-fit (red) curves (**a**) $E_{o}\left(t\right)$ and (**b**) $E_{45}\left(t\right)$ using $E_{diff}^{}\left(t\right)$ as the reference waveform.

As an example, Figure 2 shows the measured waveforms for $E_{inc}\left(t\right)$, $\tau_{as}\tau_{sa}E_{inc}\left(t\right)$, $E_{diff}^{}\left(t\right)$, $E_{o}\left(t\right)$, and $E_{45}\left(t\right)$ through an 11.54 mm thick sample of Mexican amber. The waveform $E_{diff}^{}\left(t\right)$ which was used as a reference for the deconvolution of $E_{o}\left(t\right)$ and $E_{45}\left(t\right)$ is shown in Figure 2b. For these waveforms, 60 individual waveforms were averaged. The deconvoluted waveforms $E_{o}$ and $E_{45}$ relative to $E_{diff}^{}$ as the reference are shown in Figure 3. The arrival time for any waveform deconvoluted with itself occurs at t = 80 ps. 

It is well known that the scattering of electromagnetic waves from a boundary can randomize the incident polarization. While the cross-polarization geometry of Figure 1 is designed to detect the cross-polarized waveform due to birefringence, this same geometry will also transmit scattered radiation which is cross-polarized relative to the incident polarization direction. Therefore, there are generally two contributions to the measured cross-polarization waveform: the birefringent component whose waveform shape is similar to that of Figure 2b and a cross-polarized scattering component. As the focused terahertz radiation moves closer to a boundary (e.g., the edge of the sample), the scattering component increases. However, the spot size of the focused terahertz beam on the sample is frequency-dependent, with higher terahertz frequencies forming increasingly smaller spot sizes. Therefore, as the focused terahertz beam approaches a boundary, it is the lower terahertz frequencies that first experience scattering. Their contribution to measured $E_{0}$ and $E_{45}$ deconvoluted waveforms can be minimized by slightly increasing the lower frequency bound of the Bessel function filter in the deconvolution calculation. 

The best fit curves shown in Figure 3 were calculated using the T-Ray 5000 software. The ideal waveform for the fit is generated by deconvolving the reference waveform with itself using the filters described above. The best-fit was performed over a limited time-window, as indicated by the time duration of the best-fit curves in Figure 3. The software minimized the accumulated error over the limited time window while adjusting the deconvoluted pulse amplitude and arrival time of the best-fit pulse. Since the fitting was performed over a time-window, the best-fit arrival time was significantly more accurate than the time separation (0.1 ps) between measured data points. Ideally, the measured deconvoluted pulse should be symmetric in time around its peak. However, spectral distortions due to the presence of a nearby material boundary may distort the pulse shape to be slightly asymmetric, as shown in Figure 3.

The extracted best-fit parameters from the data for the deconvoluted pulse amplitudes and arrival times *A* and Δt are given in Table 1. The error was estimated by acquiring 30 different averaged waveforms (60 individual waveforms averaged) for the $E_{inc}\left(t\right)$, $\tau_{as}\tau_{sa}E_{inc}\left(t\right)$, $E_{o}\left(t\right)$, and $E_{45}\left(t\right)$ waveforms. The pulse amplitudes and arrival times for each averaged waveform were extracted. The error was estimated to be the standard deviation of those 30 trials. The estimated error is included in Table 1. 

Using a standard propagation of errors analysis, the uncertainty in the stress direction $\sigma_{\theta }$ can be derived from Equation (2) to be

(5) $$\sigma_{\theta }^{2}=\frac{\cos^{4}2\theta }{4}\frac{E_{o}^{2}}{E_{45}^{2}}\left({\left(\frac{\sigma_{E_{o}}}{E_{o}}\right)}^{2}+{\left(\frac{\sigma_{E_{45}}}{E_{45}}\right)}^{2}\right)$$

where $\sigma_{E_{o}}/E_{o}$ and $\sigma_{E_{45}}/E_{45}$ are the relative errors in the amplitudes of the $E_{o}$ and $E_{45}$ deconvoluted waveforms, respectively. For the best-fit parameters corresponding to Figure 3 and Table 1, the best fit and uncertainty (Equation (5)) in the stress direction is θ = 36.8 ± 0.1 degrees. Following the same propagation of errors analysis, based on Equation (3), the relative error in the birefringence $\sigma_{\Delta n}/\Delta n$ is

(6) $${\left(\frac{\sigma_{\Delta n}^{}}{\Delta n}\right)}^{2}={\left(\frac{\sigma_{\Delta t_{avg}}^{}}{\Delta t_{avg}}\right)}^{2}+{\left(\frac{\sigma_{\tau_{as}\tau_{sa}E_{diff}}}{\tau_{as}\tau_{sa}E_{diff}}\right)}^{2}+\frac{\sigma_{E_{o}}^{2}E_{o}^{2}+\sigma_{E_{45}}^{2}E_{45}^{2}}{{\left(E_{o}^{2}+E_{45}^{2}\right)}^{2}}$$

For the best-fit parameters and uncertainties of Table 1, the best fit and uncertainty (Equation (6)) in the relative birefringence is $\Delta n/(n_{avg}-1)$ = (2.764 ± 0.015) × 10^−4^. 

### 3.2. Birefringence of Amber—Visible Measurements

Since amber is a photoelastic material, birefringence images acquired with visible light can assist in determining the locations of various inclusions or detect areas of varying stress induced within the amber during its creation and polymerization process. Samples of Lebanese and Mexican Chiapas amber were imaged using a polarized visible light setup. The imaging setup consisted of two film based linear polarizers placed on each side of the sample with a 6500 K light box placed on one side and a digital camera on the other. With an isotropic (non-birefringent) material placed between two polarizers whose transmission axes are orthogonal, the light which passes through the first polarizing filter maintains its polarization as it propagates through the isotropic material. Consequently, no light emerges from the second cross-polarizer, resulting in a dark image captured by the digital camera. However, if the material experiences either an applied or intrinsic stress, the induced birefringence rotates the plane of polarization of light propagating through the material resulting in a degree of transmission through the second cross-polarizing filter, resulting in a multicolored image. 

Similarly, if the amber’s birefringence properties can be approximated as a uniaxial crystal with the principal stress axes in a plane parallel to the planar faces of the sample, the incoming terahertz radiation may be propagated perpendicular to the stress axes. In this configuration, no light is transmitted through the crossed polarizers P1 and P2 unless the polarization direction of the light is modified by the birefringence. However, if the principal stress axis is either exactly parallel or perpendicular to the incident direction of polarization, then no light is transmitted; this corresponds to either θ=ϕ or θ−ϕ=π/2 in Figure 1 and is the equivalent of no transmitted light since the right-hand side of Equation (A18) vanishes under these conditions.

**Figure 4:**
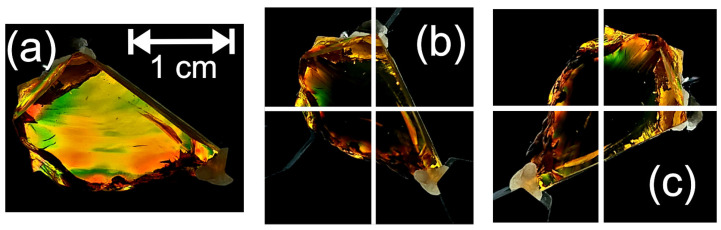
Visual birefringence images of the Lebanese amber specimen. (**a**) Crossed polarization image showing birefringence at an arbitrary angle. (**b**,**c**) Images showing minimum transmission for two orientations of the sample relative to the crossed polarizers. The transmission axes of the polarizers are parallel and perpendicular to the bottom of the page, as indicated by the white lines.

As shown in Figure 4, the Lebanese amber sample behaves as a uniaxial birefringent crystal. There are two rotational orientations of the sample (separated by 90 degrees) for which no light is transmitted. For these orientations, the polarization of the incident light is either parallel or perpendicular to the principal stress direction, resulting in no polarization rotation of the light and hence no transmission through the crossed polarizer P2. At other rotational orientations, colorful bands of light appear due to the material’s birefringence. A visualization of the birefringence in the structure using crossed polarizers is clear as the only light that reaches the camera has changed polarization due to the birefringence within the material, hence the strongest variations in color. The color variations represent the varying levels of stress within the amber. The measured rotational change between the first (Figure 4b) and second (Figure 4c) rotational angles for minimum birefringence was found to be approximately 89.5°. The measurements strongly align with the expectation that the two rotation angles corresponding to minimum polarization rotation should be 90° apart, illustrating that the two principal directions of stress in this sample are orthogonal.

**Figure 5:**
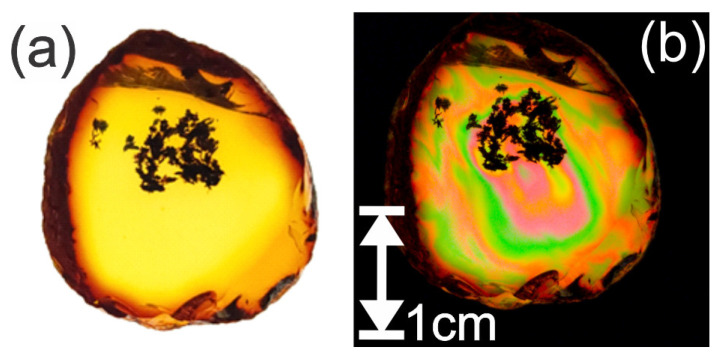
Visible polarization images due to internal stresses. Visual polarization images of Mexican Chiapas amber. (**a**) Using a vertically linear polarized light source behind the amber. (**b**) Using a linear polarized lens perpendicular (crossed) to the polarized light source.

Using this technique, areas of highly localized stress were observed in the Mexican Chiapas amber. As with the Lebanese amber, similar areas of localized birefringence were observed but with larger spatial variation within the sample. This spatial variation is denoted by the rapid change in color and contrast variation and is shown in Figure 5b. The variations in color represent the varying levels of stresses within the amber. When the sample is rotated, the color variations change depending on the angle of observation.

### 3.3. Birefringence of Amber—Terahertz Measurements

**Figure 6:**
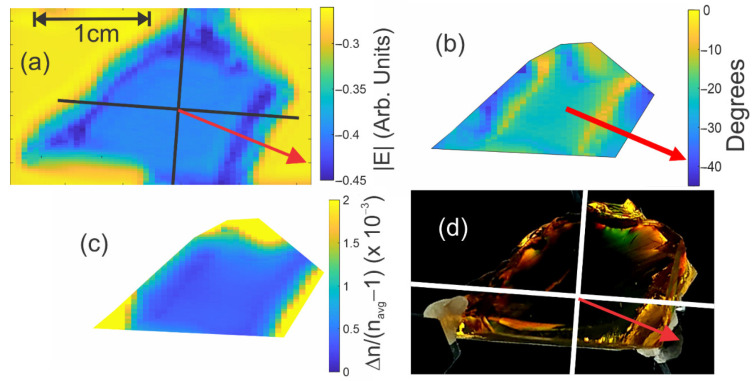
Terahertz images of Lebanese amber. (**a**) Average terahertz electric field amplitude (logarithmic scale) in the 1–2 THz frequency band. The orientation of the principal stress axis is indicated by the red arrow. (**b**) Extracted stress direction. The red arrow indicates the stress direction near the middle of the sample. (**c**) $\Delta n/(n_{avg}-1)$. (**d**) Visible image of sample from Figure 4 which has been rotated for easy comparison with the terahertz stress images.

**Figure 7:**
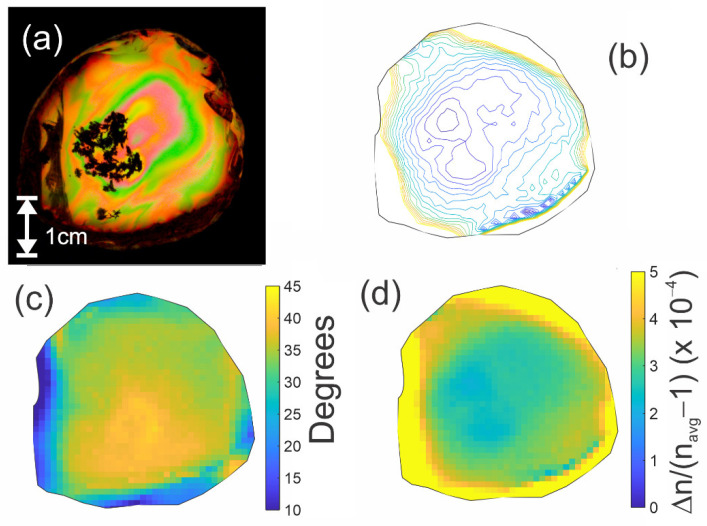
Terahertz birefringence images of Mexican amber sample. (**a**) Visible birefringence image. (**b**) Calculated contour map of stress in the terahertz range using Equation (7), as described in the text for comparison with visible stress image. The contour lines are in arbitrary units with a separation of 1 × 10^−4^ between lines. (**c**) Stress direction image. (**d**) $\Delta n/\left(n_{avg}-1\right)$.

Using the configuration of Figure 1, stress direction and relative birefringence maps were extracted using Equations (2) and (3) for the Lebanese and Mexican amber samples in the terahertz frequency range. Figure 6 shows several different terahertz images in comparison with their visible counterparts for the Lebanese amber. The transmission image in the 1–2 THz band (Figure 6a is on a logarithmic scale) can be used to identify the boundaries and orientation of the sample. Near the boundaries of the sample, increased scattering reduces the measured transmission. While data can be acquired for pixels close to the boundary of the material, the direction of the local stress as well as the magnitude of the birefringence can be distorted near the material boundary since the contribution of the cross-polarization scattering of terahertz radiation can dominate the birefringent signal. Using the edges of the boundaries defined by increased scattering in Figure 6a, a mask was generated to eliminate data that was too close to the sample boundary to be reliable. Figure 6b shows the extracted stress direction. Similar terahertz images for the Mexican amber sample are shown in Figure 7. 

### 3.4. Detection of Inclusions Using Polarized Terahertz Radiation

**Figure 8:**
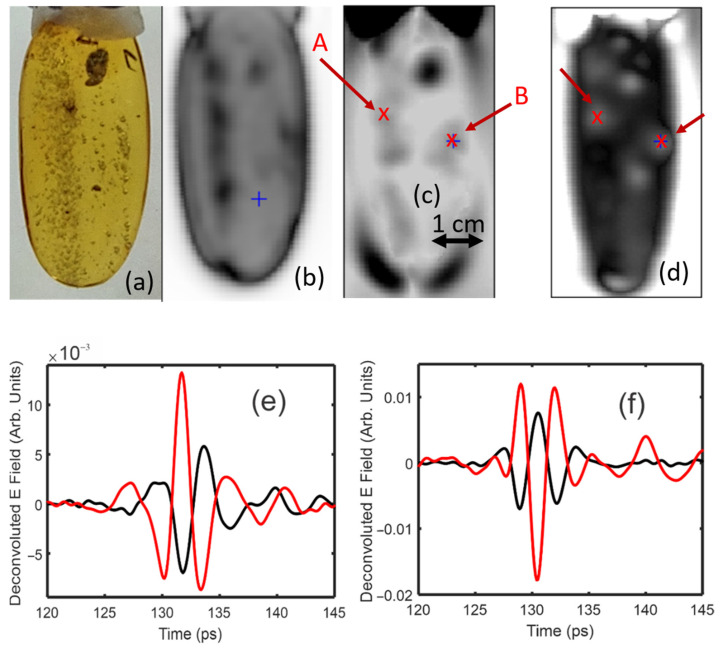
(**a**) Visible image of amber sample. (**b**) Transmitted THz power integrated from 0.5–2 THz. The image is on a logarithmic scale. (**c**) Cross-polarization deconvoluted pulse amplitude for 0 deg configuration. (**d**) Cross-polarization deconvoluted pulse amplitude for 45 deg configuration. (**e**) Deconvoluted pulses for point A. (**f**) Deconvoluted pulses for point B. The $E_{o}$ and $E_{45}$ deconvolved waveforms are denoted by the black and red curves, respectively. Note the changes in polarity of the deconvolved pulse amplitudes, indicating a significant change in the localized stress direction.

In optics, it is well known that the scattering of electromagnetic waves can change the polarization state of scattered radiation relative to the incident polarization. Using this effect, it may be possible to detect the presence of inclusions. Since it is the presence of inclusions that is of interest with regard to amber (refractive index 1.58), and not the surface topology, mineral oil (refractive index 1.46) was used as an index-matching fluid. Mineral oil was chosen for its relatively close refractive index, but also it was tested and proven to not damage these rare samples. Attempts were made to create a perfectly index-matched mineral oil solution using ceramic nanoparticles but this is not presented in this research. The sample was submerged in a plastic container filled with mineral oil. Figure 8 is shows a visible image and various terahertz images of a Dominican amber sample with various inclusions. Of particular note is the cluster of bubble inclusions. The contrast in the terahertz transmission image Figure 8b results from the scattering of terahertz from the incident beam path. In this case, the polarization orientations of the incident and detected terahertz radiation are the same. Bubble inclusions appear dark because the scattering process removes power from the transmitted beam. 

A second method for imaging inclusions is to measure a spatial map of the sample birefringence. It is well known that inclusions in an otherwise uniform material tends to concentrate the stress at the point of inclusions. Using the cross-polarization configuration of Figure 1, images of the $E_{o}$ and $E_{45}$ deconvolved pulse amplitudes are shown in Figure 8c,d. Of particular note are localized ‘hot spots’ of large polarization rotation due to birefringence, indicating a concentration of localized stress at these points. The measure of birefringence present within the amber samples around these points (×10^−4^) is two orders of magnitude larger than the birefringence measured from just the plastic box (×10^−6^) used in the experimental setup.

**Figure 9:**
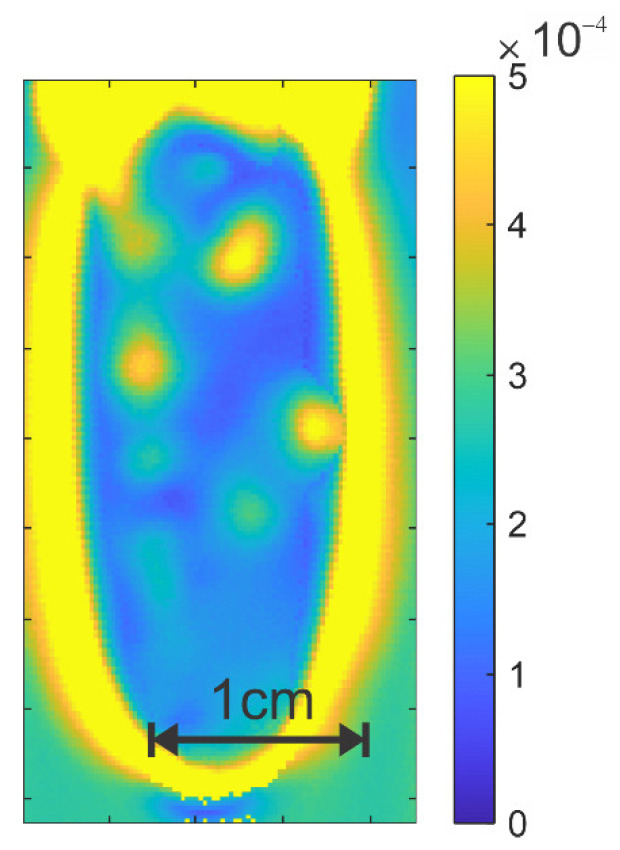
Relative birefringence map of the sample in Figure 8. Note the concentration of stress (indicated by relatively large birefringence) at points of inclusions. For specific inclusions, there is a corresponding significant change in the local stress direction (not shown).

For these two images, two points labelled A and B in the images are highlighted. The deconvoluted $E_{o}$ and $E_{45}$ waveforms corresponding to these two image locations are plotted in Figure 8e,f. The pulse amplitudes at location B are inverted compared to those at location A, indicating a significant change in the localized stress direction. Analysis of the changing deconvolved pulse amplitudes using Equations (2) and (3) yielded the relative birefringence map shown in Figure 9.

### 3.5. Terahertz-Computed Tomography Imaging of Inclusions

In this section, the prospect of using THz-CT to image inclusions in 3D is explored. A simple method to minimize the artifacts from beam refraction is to immerse the sample in an index-matching material. The amber sample was mounted to the end of a rotation stage and immersed into mineral oil within a plastic container for imaging [43].

**Figure 10:**
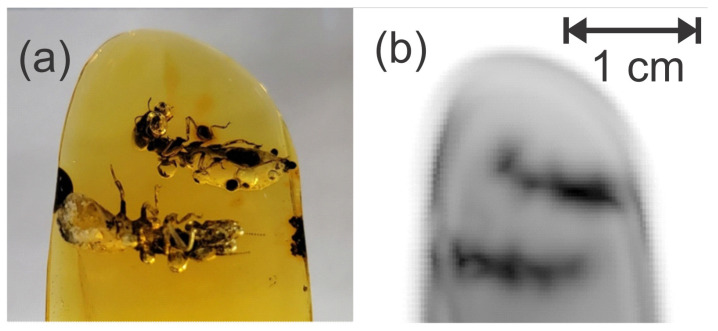
(**a**) Closeup reference picture of the two termite workers in the Dominican amber matrix. (**b**) Terahertz image generated from the natural log of the mean terahertz transmitted amplitude in the 0.5–2 THz band.

**Figure 11:**
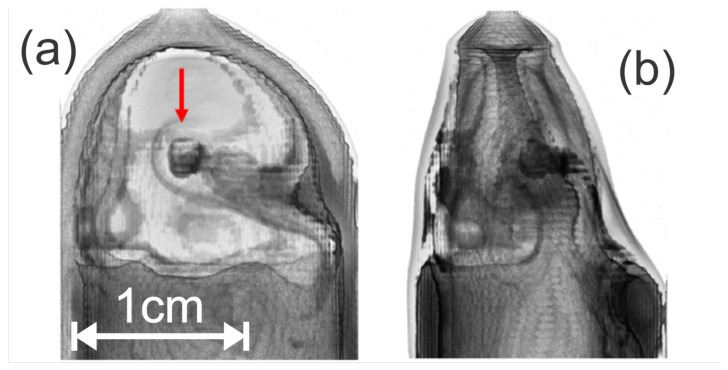
3D reconstruction of the Dominican amber sample based on transmission amplitude with optimized luminance, allowing for internal structure observation. (**a**) Front profile with red arrow indicating termite head location. (**b**) Side profile. An animated GIF file of the 3D reconstructed image is included as a Supplementary Materials Video S1.

Transmitted terahertz waveforms were acquired on a pixel-by-pixel basis for each rotational position of the sample. The 2D images (Figure 10) were acquired with a 0.25 mm pixel size. The images were acquired for rotational increments of every 3 degrees for a full 360-degree rotation. Using the transmission images, sinograms and tomographic images were created using a custom MATLAB code [43]. The tomographic images were turned into a 3D reconstruction of the amber, shown in Figure 11, using the Volume Viewer plugin in ImageJ [44]. This plugin allows for adjustments to the reconstruction’s alpha values based on multidimensional transfer functions. In an attempt to image the inclusions separately, and by using transmission as the pixel value in the image reconstruction, more of the internal structure was able to be imaged by optimizing the luminance using a 1D transfer function within the 3D reconstruction.

### 3.6. Detection of Termite Inclusions and Air Bubbles Using Polarized Terahertz Radiation

Following the same procedure as detailed above, cross-polarization imaging was utilized to image termite inclusions and air bubbles, consistent with methane, in the Dominican amber. As with the previous sample in Figure 8, the termite inclusions tended to concentrate the intrinsic strain in the sample.

**Figure 12:**
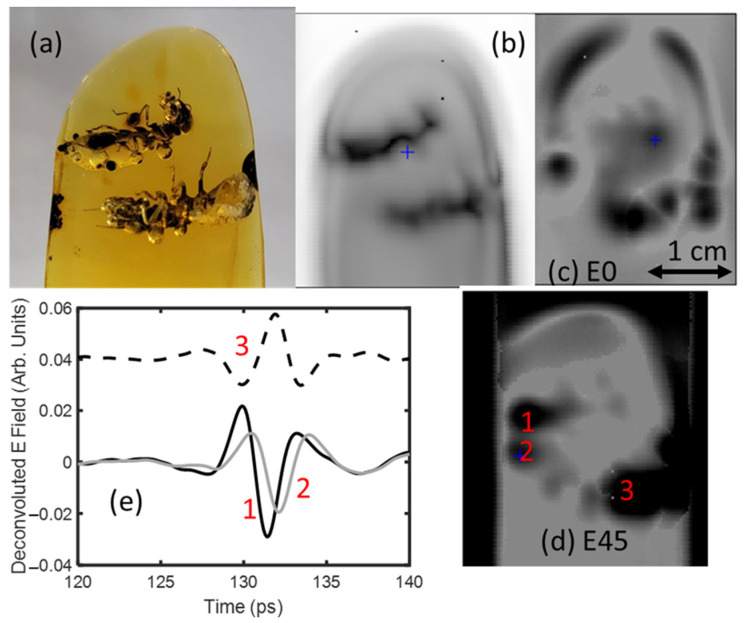
(**a**) Visible image (through mineral oil) of termite inclusions in Dominican amber. (**b**) Logarithm of the transmitted terahertz electric field amplitude in the 0.5–2 THz range. (**c**) Deconvoluted pulse amplitude of $E_{0}$. (**d**) Deconvoluted pulse amplitude of $E_{45}$. (**e**) Deconvoluted pulse shape for positions 1, 2, and 3, as denoted in (**d**).

Figure 12b shows a transmission image for the average detected power in the 0.5–2 THz frequency band. Figure 12c,d show images of the $E_{o}$ and $E_{45}$ amplitudes, respectively. There are localized regions, of similar size and shape to the termites/bubbles, in which the birefringence is relatively large compared to the surrounding inclusion-free regions of amber. The $E_{45}$ waveforms for three distinct locations show an inversion of the pulse shape at position 3 relative to positions 1 and 2, indicating a localized change in stress direction due to the inclusions within the 30 mm^2^ amber matrix surrounding the termite. This inversion of pulse shape is shown in Figure 12e, with the third pulse location shifted for visibility.

## 4. Discussion

Note that the stress angle in Figure 6 is uniform in the central portion of the sample where distortions of the $E_{o}$ and $E_{45}$ waveforms are minimized since the pixels are relatively far away from the sample boundary. A map of the relative birefringence magnitude is shown in Figure 6c. Throughout the central portion of the sample, the birefringence is relatively uniform. In comparing the stress direction inferred by the terahertz measurements (Figure 6b) to the orientation inferred by visible measurements (Figure 6d), one may conclude that there is good agreement between the visible and terahertz stress birefringence measurements. This supports the notion that while the electromagnetic frequencies in the visible and terahertz ranges are roughly three orders of magnitude different, the manifestation of birefringence due to intrinsic stress is the same.

Similar terahertz images for the Mexican amber sample are shown in Figure 7. An interesting feature of the internal stresses of this sample is that the stress direction appears to spatially vary over the central portion of the sample from approximately 34–40 degrees while the value of $\Delta n/\left(n_{avg}-1\right)$ varies from 3 × 10^−4^ to 4 × 10^−4^. Analysis of the uncertainty in the extracted values described above indicates that these variations are larger than the uncertainty and represent spatial variations in the stress direction and magnitude. 

In order to compare the visible photoelasticity image with the terahertz image, the terahertz data can be processed to create an equivalent image to the visible image. Starting from Equation (A19) in the Appendix A, the measured electric field through crossed polarizers may be written as

(7) $$E_{RX}=\frac{\tau_{as}\tau_{sa}}{c_{o}t_{o}}\left[\Delta n\;L\;E_{diff}\right]\left(\sin\left(\theta +\theta_{o}\right)\cos\left(\theta +\theta_{o}\right)\right),$$

for which $\theta_{o}$ is the rotational offset between the coordinate systems of the visible and terahertz measurements and θ is the measured stress angle in the terahertz image. 

A terahertz photoelastic image (Figure 7b) that mimics the visible photoelastic image of Figure 7a was generated according to Equation (7) on a pixel-by-pixel basis by multiplying images of the relative birefringence (Figure 7d), the time delay through the sample (which is a measure of the thickness, *L*, of the sample), and the $\sin(\theta +\theta_{o})\cos(\theta +\theta_{o})$ factor, which was determined by the stress direction (Figure 7c). The terahertz power is proportional to the square of Equation (7). The resulting image was plotted in Figure 7b as a contour plot. When comparing that image with Figure 7a, it is possible to note that the contour lines of the detected terahertz power are similar in shape to the visible color band shapes of Figure 7a, indicating qualitative agreement between visible and terahertz birefringence images. This suggests that terahertz birefringence measurements can extend stress mapping to materials which are opaque to visible light (such as most plastics and certain sources of amber).

As shown in Figure 11, the reconstruction with the adjusted luminance values allows for the visualization of different parts of the internal structure of the amber specimen. A full 3D image reconstruction is available in the Supplemental Materials. The lower termite has been completely lost due to the high amount of refraction at that location. This is due to a combination of a mismatch in the refractive index between the mineral oil and amber, as well as the rounded, non-parallel shape of the amber creating a non-normal reflection at that location, seen in the side profile reconstruction (Figure 11b). The alpha value adjustment does show a partial reconstruction of the upper termite, specifically the head, that may be caused by the increased density within. The head capsule of insects, and in particular termites, are more heavily sclerotized than other segments, leading to greater cuticle (exoskeleton) thickness as well as potential metal sequestration [45,46]. There is a lighter region of decreased refraction in the upper portion of the sample surrounding the upper termite due to the parallel faces of the sample within that region, allowing for close-to-normal incidence reflection measurements.

Insect inclusions in amber are heterogeneous in their internal composition. Entombed insects occasionally retain elements of internal soft tissue; however, it is more common for insect exoskeletons to be essentially hollow. Soft tissue that is degraded by microbes during the resin-capture stage is effectively replaced by air, leaving void space between the exoskeleton [29]. Such void spaces are likely to contribute significant strain within the amber matrix. Resin may also seep into void spaces within insect cuticles—such infilling is likely contingent on the viscosity of the resin as well as the level of exoskeleton degradation. Consequently, the internal composition and subsequent strain impact of inclusions can vary from insect to insect, even within the same sample.

## 5. Conclusions

Using terahertz radiation transmission in a cross-polarization geometry, stress maps for the normalized birefringence and stress direction may be obtained nondestructively. The analysis method utilizes a deconvolution method to determine the arrival times and amplitude of the cross-polarized terahertz pulses through a birefringent material. Using amber as a material of interest, the stress maps show that Lebanese amber samples with no inclusions behave as classic uniaxial birefringent (photoelastic) materials whose inferred stress axes in the terahertz spectral range agree well with visible photoelasticity measurements. Moreover, the terahertz data can be processed into stress contour maps which closely follow the color bands of visible photoelasticity measurements in Mexican amber.

The cross-polarization experimental configuration enables stress levels within the amber matrix to be visualized while also outlining highly localized regions of stress surrounding inclusions. Scattered cross-polarization radiation, while an important contrast mechanism for the reduction in transmitted power, enabling the visualization of air bubble inclusions within amber, can be separated from the photoelastic response. Birefringence stress maps clearly show localized increases in stress magnitude and direction located at points of inclusions.

A methodology for creating 3D reconstructions of amber samples was created using a refractive index matching material. With further investigation into this approach, one should be able to extract a 3D reconstruction of inclusions independently from the surrounding amber matrix. This would allow for the study of inclusions’ shapes, sizes, etc. 

## Figures and Tables

**Table 1:** Extracted amplitudes and arrival times (in ps) for the various deconvoluted time-domain waveforms of Figure 3. The deconvolved pulse time is measured relative to the arrival time of $E_{inc}$.

Waveform	Deconvoluted Amplitude (Arb. Units)	Deconvoluted Pulse Time (ps)
$\tau_{as}\tau_{sa}E_{inc}^{}$	0.4127 ± 0.0006	21.94 ± 0.003
$E_{o}$	(1.200 ± 0.012) × 10^−2^	
$E_{45}$	−(0.3545 ± 0.012) × 10^−2^	

## Data Availability

The data presented in this study are available on request from the corresponding author.

## Supplementary Materials

The following supporting information can be downloaded at: https://www.mdpi.com/article/10.3390/polym14245506/s1, Video S1: Terahertz CT Image of Termites.

## Appendix A

For simplicity, it is assumed that the sample can be treated as a uniaxial birefringent material in which the principal stress axes are perpendicular to the propagation direction of the terahertz waves. The electric field after transmission through polarizer P1 in Figure 1 can be expressed as

(A1) $$E_{TX}=E_{inc}\left(t\right)\left(\hat{x}\cos\phi +\hat{y}\sin\phi \right)$$

where ϕ is the angle between the horizontal x-axis and the transmission axis of polarizer P1. As the terahertz pulse enters the sample, the vector components of the electric field are written in terms of the basis of the fast (lower refractive index) and slow (higher refractive index) stress axes of the material, with the fast axis oriented at an angle θ with respect to the horizontal x axis:

(A2) $$\hat{x}={\hat{\sigma }}_{1}\cos\theta -{\hat{\sigma }}_{2}\sin\theta$$

(A3) $$\hat{y}={\hat{\sigma }}_{1}\sin\theta +{\hat{\sigma }}_{2}\cos\theta$$

The electric field, just as it enters the sample, using Equations (A1)–(A3) can be expressed as

(A4) $$E_{s\_in}\left(t\right)=E_{inc}\left(t\right)\tau_{as}\left[{\hat{\sigma }}_{1}\left(\cos\phi \cos\theta +\sin\phi \sin\theta \right)+{\hat{\sigma }}_{2}\left(\sin\phi \cos\theta -\cos\phi \sin\theta \right)\right]$$

where $\tau_{as}$ is the transmission coefficient in terms of propagating from air into the material. Since the electric field is expressed in terms of the basis for the fast and slow axes, the additional time delay for propagation through the sample and back into the air can be expressed as

(A5) $$E_{s\_out}\left(t\right)=\tau_{as}\tau_{sa}\left[{\hat{\sigma }}_{1}E_{inc}\left(t+\Delta t_{\sigma_{1}}\right)\left(\cos\phi \cos\theta +\sin\phi \sin\theta \right)+{\hat{\sigma }}_{2}E_{inc}\left(t+\Delta t_{\sigma_{2}}\right)\left(\sin\phi \cos\theta -\cos\phi \sin\theta \right)\right]$$

where the propagation times for the two principal axes in the material are given by $\Delta t_{\sigma_{1}}=n_{\sigma_{1}}L/c_{o}$ and $\Delta t_{\sigma_{2}}=n_{\sigma_{2}}L/c_{o}$, where $c_{o}$ represents the speed of light in a vacuum while $n_{\sigma_{1}}$ and $n_{\sigma_{2}}$ denote the refractive indices along the corresponding principal stress axes. The product $\tau_{as}\tau_{sa}$ accounts for any losses in transmission due to the reflection of a portion of the terahertz pulse at the air–sample interfaces.

After propagating the terahertz pulse through the sample, one translates Equation (A5) back into the laboratory x–y frame using

(A6) $${\hat{\sigma }}_{1}=\hat{x}\cos\theta +\hat{y}\sin\theta$$

(A7) $${\hat{\sigma }}_{2}=-\hat{x}\sin\theta +\hat{y}\cos\theta$$

Finally, the resulting electric field through the polarizer P2 is calculated by taking the dot product with the transmission axis polarization of P2 given by $\hat{P}2=-\hat{x}\sin\phi +\hat{y}\cos\phi$. After rearranging terms, the final electric field detected by the receiver can be expressed as

(A8) $$E_{RX}\left(t\right)=\tau_{as}\tau_{sa}\left[E_{inc}\left(t+\Delta t_{\sigma_{1}}\right)-E_{inc}\left(t+\Delta t_{\sigma_{2}}\right)\right]\left(\sin\theta \cos\theta \left(\cos^{2}\phi -\sin^{2}\phi \right)+\sin\phi \cos\phi \left(\sin^{2}\theta -\cos^{2}\theta \right)\right)$$

As a quick check, when there is no birefringence, then $\Delta t_{\sigma_{1}}=\Delta t_{\sigma_{2}}$ and the detected electric field vanishes. Likewise, if θ=ϕ, then the polarizers P1 and P2 are along the principal stress and birefringence axes, yielding no detectable electric field since for this orientation of the sample relative to the polarizers, the birefringence does not rotate the polarization of the incident terahertz pulse.

The next step is to evaluate the difference in the two time-shifted electric fields $E_{inc}\left(t+\Delta t_{\sigma_{1}}\right)-E_{inc}\left(t+\Delta t_{\sigma_{2}}\right)$. To facilitate the calculation, Fourier transforms, as defined below, are utilized

(A9) $$f\left(\nu \right)=\int_{-\infty }^{\infty }f\left(t\right)\;e^{-i2\pi \nu t}dt$$

(A10) $$f\left(t\right)=\int_{-\infty }^{\infty }f\left(\nu \right)\;e^{i2\pi \nu t}d\nu$$

If one assumes that the refractive indices along the principal stress directions are constant as a function of frequency, the Fourier transform of the time-shifted incident electric field may be evaluated as

(A11) $$E_{\sigma_{1}}\left(\nu \right)=\int_{-\infty }^{\infty }E_{inc}\left(t+\Delta t_{\sigma_{1}}\right)\;e^{-i2\pi \nu t}dt=E_{inc}\left(\nu \right)e^{i2\pi \nu \;\Delta t_{\sigma_{1}}}$$

This result is expected because in the frequency domain, the time delay is manifested as a simple multiplication by a phase factor. Using Equation (A11), the difference in the time-shifted electric fields of Equation (A8) can be evaluated as

(A12) $$E_{inc}\left(t+\Delta t_{\sigma_{1}}\right)-E_{inc}\left(t+\Delta t_{\sigma_{2}}\right)=\int_{-\infty }^{\infty }\left(E_{\sigma_{1}}\left(\nu \right)-E_{\sigma_{2}}\left(\nu \right)\right)\;e^{i2\pi \nu t}d\nu$$

Factoring out the average phase shift on the right-hand side of the equation gives

(A13) $$E_{inc}\left(t+\Delta t_{\sigma_{1}}\right)-E_{inc}\left(t+\Delta t_{\sigma_{2}}\right)=\int_{-\infty }^{\infty }E_{inc}\left(\nu \right)e^{i2\pi \nu \left(\Delta t_{\sigma_{2}}+\Delta t_{\sigma_{1}}\right)/2}\left(e^{i2\pi \nu \left(\Delta t_{\sigma_{1}}-\Delta t_{\sigma_{2}}\right)/2}-e^{-i2\pi \nu \left(\Delta t_{\sigma_{1}}-\Delta t_{\sigma_{2}}\right)/2}\right)\;e^{i2\pi \nu t}d\nu$$

(A14) $$=-\int_{-\infty }^{\infty }2iE_{inc}\left(\nu \right)e^{i2\pi \nu \left(\Delta t_{\sigma_{2}}+\Delta t_{\sigma_{1}}\right)/2}\sin\left(\pi \nu \;\Delta n\;L/c_{o}\right)\;e^{i2\pi \nu t}d\nu$$

where the birefringence $\Delta n=\left(n_{\sigma_{2}}-n_{\sigma_{1}}\right)L/c_{o}$. In the event that the birefringence is small such that $\pi \nu \;\Delta n\;L/c_{o}\ll 1$, the sine function can be approximated such that

(A15) $$E_{inc}\left(t+\Delta t_{\sigma_{1}}\right)-E_{inc}\left(t+\Delta t_{\sigma_{2}}\right)≃-2\pi i\frac{L\;\Delta n}{c_{o}}\int_{-\infty }^{\infty }\nu E_{inc}\left(\nu \right)e^{i2\pi \nu \left(\Delta t_{\sigma_{2}}+\Delta t_{\sigma_{1}}\right)/2}\;e^{i2\pi \nu t}d\nu$$

(A16) $$≃-\frac{\Delta n\;L}{c_{o}}\frac{d}{dt}\left[E_{inc}\left(t+\frac{\Delta t_{\sigma_{2}}+\Delta t_{\sigma_{1}}}{2}\right)\right]=\frac{\Delta n\;L}{c_{o}}\underset{\Delta t_{0}\to 0}{\lim}\left[\frac{E_{inc}\left(t+\frac{\Delta t_{\sigma_{2}}+\Delta t_{\sigma_{1}}}{2}\right)-E_{inc}\left(t+\Delta t_{0}+\frac{\Delta t_{\sigma_{2}}+\Delta t_{\sigma_{1}}}{2}\right)}{\Delta t_{0}}\right]$$

where on the right-hand side of Equation (A16), the time derivative of the incident electric field pulse is approximated as the finite difference. This equation justifies the following model for the difference in the time-delayed incident pulses

(A17) $$E_{inc}\left(t+\Delta t_{\sigma_{1}}\right)-E_{inc}\left(t+\Delta t_{\sigma_{2}}\right)≃\Delta n\;A\;E_{diff}\left(t\right)$$

where Δn is the material birefringence, $E_{diff}\left(t\right)=E_{inc}\left(t+\left(\Delta t_{\sigma_{2}}+\Delta t_{\sigma_{1}}\right)/2\right)-E_{inc}\left(t+\Delta t_{0}+\left(\Delta t_{\sigma_{2}}+\Delta t_{\sigma_{1}}\right)/2\right)$, and $A=L/c_{o}\Delta t_{o}$ is a scaling factor. The differential electric field pulse $E_{diff}$ is generated as described in the results section.

Combining Equations (A8) and (A16) gives the detected terahertz time-domain waveform as

(A18) $$E_{RX}\left(t\right)=\tau_{as}\tau_{sa}\left[\Delta n\;A\;E_{diff}\left(t\right)\right]\left(\sin\theta \cos\theta \left(\cos^{2}\phi -\sin^{2}\phi \right)+\sin\phi \cos\phi \left(\sin^{2}\theta -\cos^{2}\theta \right)\right).$$

The goal of the terahertz birefringence measurement is to measure the detected terahertz waveform at two different ϕ values to uniquely determine the birefringence magnitude, Δn, and the stress principal stress direction, θ, of the material. Evaluating the measured time-domain waveform for the polarizer P1 making angles of ϕ = 0° and ϕ = 45° with respect to the x-axis (polarizer P2 is orthogonal to P1 for both ϕ values) gives two equations for the measured waveforms

(A19) $$E_{0}\left(t\right)=\tau_{as}\tau_{sa}\left[\Delta n\;A\;E_{diff}\left(t\right)\right]\left(\sin\theta \cos\theta \right)$$

(A20) $$E_{45}\left(t\right)=\tau_{as}\tau_{sa}\left[\Delta n\;A\;E_{diff}\left(t\right)\right]\left(\sin^{2}\theta -\cos^{2}\theta \right)/2$$

By extracting the pulse amplitudes of the waveforms denoted by $E_{0}$ and $E_{45}$, the stress direction

(A21) $$\frac{E_{o}}{E_{45}}=\frac{2\sin\theta \cos\theta }{\sin^{2}\theta -\cos^{2}\theta }=-\tan2\theta$$

is calculated by dividing Equations (A19) and (A20). This is the same result as derived by [38] using a Jones matrix approach.

The magnitude of the birefringence can be determined by measuring the amplitudes of the waveforms denoted by $E_{0}$, $E_{45}$, and $E_{diff}$. Equations (A19) and (A20) may be squared and added to yield

(A22) $$\Delta n=\frac{2c_{o}\Delta t_{o}}{L}\sqrt{{\left(\frac{E_{o}}{\tau_{as}\tau_{sa}E_{diff}}\right)}^{2}+{\left(\frac{E_{45}}{\tau_{as}\tau_{sa}E_{diff}}\right)}^{2}}$$

Note that the definition of $\tau_{as}\tau_{sa}E_{diff}$ in Equation (A22) takes into account the reflective losses of the incident electric field pulse as it propagates through the air–sample interfaces. An added benefit of measuring the time shifts $\Delta t_{\sigma_{1}}$ and $\Delta t_{\sigma_{2}}$ referenced to no sample in the terahertz beam path is that it enables one to normalize out the thickness from Equation (A22). In this case, the average of the time shifts may be written as

(A23) $$\frac{\Delta t_{\sigma_{1}}+\Delta t_{\sigma_{2}}}{2}=\frac{L}{c_{o}}\left(\left(\frac{n_{\sigma_{1}}+n_{\sigma_{2}}}{2}\right)-1\right)=\frac{L}{c_{o}}\left(n_{average}-1\right)$$

Dividing Equation (A22) by Equation (A23) gives the relative birefringence as

(A24) $$\frac{\Delta n}{n_{average}-1}=2\frac{\Delta t_{o}}{\left(\Delta t_{\sigma_{1}}+\Delta t_{\sigma_{2}}\right)/2}\sqrt{{\left(\frac{E_{o}}{\tau_{as}\tau_{sa}E_{diff}^{}}\right)}^{2}+{\left(\frac{E_{45}}{\tau_{as}\tau_{sa}E_{diff}^{}}\right)}^{2}}$$

The evaluation of Equation (A24) requires the measurement of the amplitudes and arrival times of the three waveforms denoted by $E_{o}$, $E_{45}$, and $\tau_{as}\tau_{sa}E_{inc}$.
