# Supplementary material for: Evaluation of Fossil Amber Birefringence and Inclusions Using Terahertz Time-Domain Spectroscopy
Source: Polymers (Basel). 2022 Dec 15;14(24):5506. doi: 10.3390/polym14245506 (PMC9780848; doi:10.3390/polym14245506)
Supplement: Supplementary file 1 [file polymers-14-05506-s001.zip › polymers-2015269-supplementary.docx]

**Supplementary Materials**

Evaluation of Fossil Amber Birefringence and Inclusions Using Terahertz Time-Domain Spectroscopy

Alexander T. Clark ^1^, Sophia D’Anna ^1^, Jessy Nemati ^1^, Phillip Barden ^2,3^, Ian Gatley ^1^ and
John Federici ^1,^*

^1^ Department of Physics, New Jersey Institute of Technology, Newark, NJ 07102, USA

^2^ Federated Department of Biological Sciences, New Jersey Institute of Technology, Newark, NJ 07102, USA

^3^ Division of Invertebrate Zoology, American Museum of Natural History,
New York, NY 10024, USA

***** Correspondence: federici@njit.edu


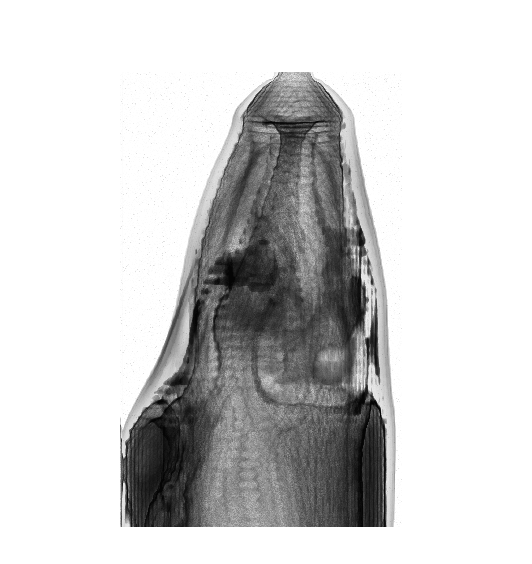


**Video S1: Terahertz CT Image of Termites.**
